# Research on the Quality of Composite Pipe Components in Fluid-Powered Projectile-Assisted Injection Molding

**DOI:** 10.3390/polym17040489

**Published:** 2025-02-13

**Authors:** Tangqing Kuang, Chuixin Kong, Hesheng Liu, Fan Yang

**Affiliations:** 1 School of Mechatronics & Vehicle Engineering, East China Jiaotong University, Nanchang 330013, China; tkuang@ecjtu.edu.cn; 2 School of Materials Science & Engineering, East China Jiaotong University, Nanchang 330013, China; kcx17852535783@163.com; 3 Center for Basic Experiments and Engineering Practice, East China Jiaotong University, Nanchang 330013, China; fyang@ecjtu.edu.cn

**Keywords:** fluid-projectile-assisted co-injection molding, composite pipe fittings, apparent quality, pressure resistance performance

## Abstract

Composite pipe fittings with an outer layer of 20% long glass fiber-reinforced polypropylene (LGFR-PP) and an inner layer of polypropylene (PP) were prepared via water-powered projectile-assisted co-injection molding short-shot (W-PACIM-S), water-powered projectile-assisted co-injection molding overflow (W-PACIM-O), gas-powered projectile-assisted co-injection molding short-shot (G-PACIM-S), and gas-powered projectile-assisted co-injection molding overflow (G-PACIM-O)techniques. The effects of different injection molding processes on the wall thickness, inner surface roughness, glass fiber orientation, and pressure resistance of pipe fittings were studied to evaluate the quality of the pipe fittings formed by each process. Compared with the short-shot method, the overflow method results in pipes with thinner walls in each layer, a more uniform distribution, smoother inner wall surfaces, and better orientation of glass fibers along the axial direction in the near boundary layer, resulting in better pressure resistance. Under the same injection method, the difference in fluid medium did not significantly change the trend of wall thickness variation in each layer. However, compared with gas, high-pressure water improves the uniformity of the pipe wall thickness and inner wall quality. In addition, the introduction of the warhead is more conducive to improving the degree of glass fiber orientation of the pipe fittings, and the thickness of the residual wall thickness of the pipe fittings has a great influence on the pressure resistance of the pipe fittings.

## 1. Introduction

**Figure 1:**
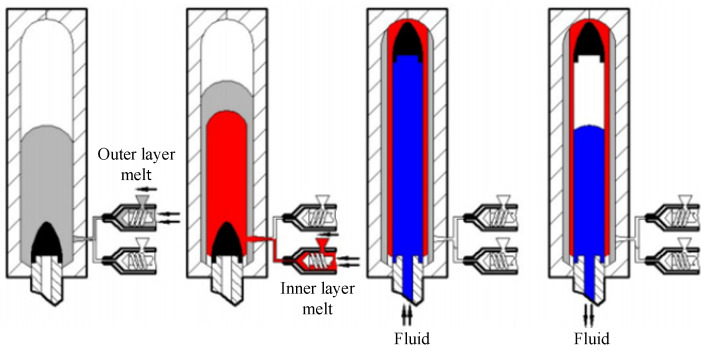
Forming process of the F-PACIM method process. (gray represents the outer melt, red represents the inner melt, blue represents the fluid, and black represents the bullet).

In recent years, with the gradual implementation of national environmental policies, various industries have put forward higher requirements for the multifaceted performance of injection-molded products [1,2]. In this context, long glass fiber-reinforced polypropylene (LGFR-PP), a representative modified material, has been widely used because of its excellent mechanical properties, good corrosion resistance, and electrical insulation properties [3,4]. Injection molding is a widely used process in polymer processing. Fluid-assisted co-injection molding (FACIM) involves sequentially injecting inner and outer melt layers under the driving force of high-pressure fluid, forming hollow pipes with complex structures. On this basis, researchers have developed a new injection molding process—fluid-powered projectile-assisted co-injection molding (F-PACIM)—which introduces fixed-size pellets to control the uniformity of part wall thickness and conserve materials [5,6,7]. According to the classification of fluid media used, FPACIM can be divided into water-powered projectile-assisted co-injection molding (W-PACIM) and gas-powered projectile-assisted co-injection molding (G-PACIM), each of which can be divided into two methods on the basis of whether the inner and outer layers of the melt are filled in the mold cavity: the short-shot method and overflow method. The process flow is shown in Figure 1: First, place the projectile into the nozzle, close the mold, and then inject the inner and outer melt into the mold cavity in sequence. Then, the gas pushes the projectile through the melt to form a composite pipe. Finally, release the pressure and discharge the fluid.

On the basis of the advantages of fluid-assisted injection molding technology, many scholars have researched the quality and processing methods of parts. Zhang [8,9] conducted experimental research on the factors influencing water-assisted injection molding (WAIM) on glass fiber orientation and the effect of the overflow short jet water-assisted injection molding process on the quality of short glass fiber reinforced polypropylene (SGFR-PP) pipe fittings. Ding et al. [10] compared the residual wall thickness distribution of W-PAIM products through experiments and numerical simulations. The results showed that compared with WAIM, W-PAIM can effectively control the wall thickness of products. Hopmann et al. [11] studied the material applicability of W-PAIM, and the results showed that the inner wall of the pipe formed by EPDM rubber was smoother, while the wall thickness of the pipe formed by nitrile rubber was more uniform. Zhong and Wang et al. [12,13] used experimental methods to explore the effects of different processing methods on bent and straight pipes and reported that the wall thickness of pipe fittings manufactured via gas-assisted injection molding (GAIM) was the thickest, whereas the uniformity was the worst. Introducing projectiles can increase the orientation of the fiberglass along the axial direction of the pipe but may exacerbate fiberglass fracturing. The application of additive manufacturing (3D printing) technology in downstream key industries is extensive and in-depth, benefiting various key industries such as molds, aerospace and automotive manufacturing, medical devices, machine tools, and consumer electronics. This technology not only improves manufacturing efficiency and accuracy but also promotes innovation and development in various industries. With the further maturity of technology and the expansion of applications, 3D printing technology will demonstrate its unique value in more fields in the future [14]. However, current research on the impact of composite pipe manufacturing processes on the apparent quality and mechanical properties of products is relatively limited. At present, there is relatively little research on the comparative analysis of the FPACIM process, and the quality of the inner wall surface molding is mostly analyzed by visual observation. This article selects four process methods that can accurately meet the specific needs of different products and have important value in practical production. It explores the impact and mechanism of F-PACIM on the quality of composite pipe fittings, providing theoretical reference for the application of polymer composite materials in industrial production.

## 2. Experimental Procedure

### 2.1. Materials

**Figure 2:**
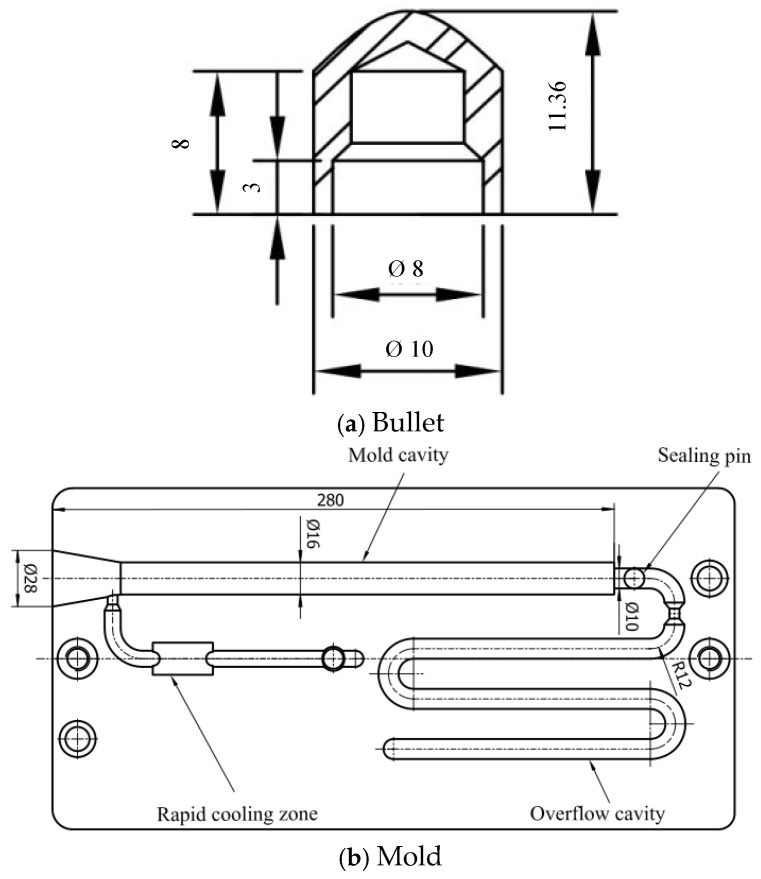
Schematic diagram of structure (all dimensions are in millimeters).

The outer layer polymer resin used in this work was 20 wt% commercially available grade GB302HP LGFR-PP from the Saudi Basic Industries Corporation. Its average glass fiber length is approximately 12 mm, the melt flow index is 5 g/10 min, and the heat deformation temperature is 158 °C. The inner layer material was PP produced by the China Petroleum and Chemical Corporation with a melt flow index of 3 g/10 min and a heat deformation temperature of 91 °C. The bullet material resin used was Nylon 6 produced by the Taiwan Jisheng Company (New Taipei City, Taiwan), shaped as shown in Figure 2a. Its melt index is 10.5 g/10 min; the hot deformation temperature is 160 °C.

### 2.2. Experimental Platform

The experiments were carried out on a lab-developed gas/water co-injection molding experimental platform, which comprised a twin-screw injection molding machine (MA1600M/E type, produced by Haitian Plastic Machinery Group Co., Dongguan, China), a fluid injection mold, a water injection system [15], a gas injection system [16,17], a mold temperature machine(BTM-09M model, produced by Shenzhen Borui Ke Machinery Co., Shenzhen, China), a 3D scan contometer (MA1600M/E type, produced by the Haitian Plastic Machinery Group Co., Ningbo, China) a scanning electron microscope (SEM) (A Nova None SEM 450 model produced by the FEI Company in the Netherlands, Eindhoven, The Netherlands), a pipe pressure testing machine(MTSH-06 type, produced by the Tianjin Metes Testing Machine Factory, Tianjin, Chian). To simplify the analysis and ignore the effects of flow direction and cavity cross-sectional changes on the formation of pipe fittings, a cavity with a diameter of 16 mm is selected, which references structural diagram Figure 2b created by the research group.

### 2.3. Experimental Scheme

On the basis of an experimental platform previously built by the Hesheng Liu research group [9,10,11], composite pipe fittings were prepared via the G-PACIM-S, W-PACIM-S, W-PACIM-O, and G-PACIM-O processes. The injection molding process parameters were determined on the basis of previous research by our research group [12], as shown in Table 1.

### 2.4. Testing and Characterization

#### 2.4.1. Measuring the Wall Thickness and Uniformity of Pipes

**Figure 3:**
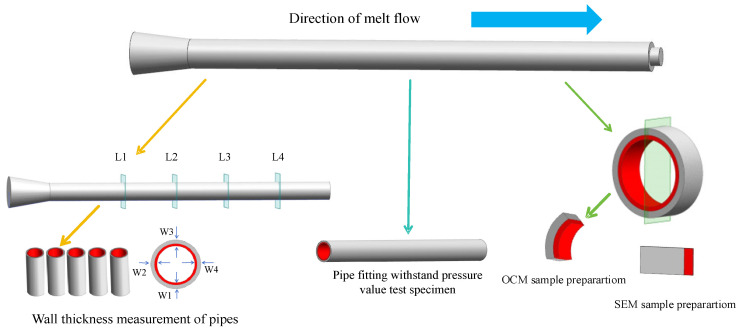
Specimen preparation process.

To ensure the accuracy of the experimental data, a small amount of coloring agent is added to the inner melt before injection, making the inner layer of the pipe appear red while the outer layer maintains its original color. As shown in Figure 3, four equal points (L1, L2, L3, and L4) are set on the straight pipe, and four equal measurement points (W1, W2, W3, and W4) are set on the cross-section cut horizontally. The arithmetic mean of the wall thickness at four points in each direction is taken. The total wall thickness and inner wall thickness are measured separately, and the outer wall thickness is calculated on the basis of the difference between the two.

The standard deviation represents the degree of dispersion between the data points and the mean. The larger the standard deviation is, the greater the difference between the pipe wall thickness value and the average wall thickness value. In contrast, the smaller the standard deviation is, the closer the wall thickness of the pipe fitting is to the average value.

#### 2.4.2. Preparation of Rough Samples

To ensure that the observation surface is as flat as possible, as shown in Figure 3, a 150 mm long circular ring is cut at the middle position of the pipe fitting and horizontally from the 1/3 position of the cross-sectional diameter. Then, it is placed on the stage for observation.

#### 2.4.3. Preparation of SEM Samples

Observation samples: A 6 mm wide circular ring was cut in the middle of the pipe fitting, cut in half along the axial direction, and placed in liquid nitrogen for 120 min to split thin slices from the brittle surface. The cross-section is fixed upward on the stage for gold spraying treatment and observed with an SEM. The preparation process is shown in Figure 3.

#### 2.4.4. Preparation of Pressure-Resistant Test Samples

The top and bottom of the pipe fittings may be affected by the overflow chambers and nozzles. To ensure the accuracy of the experimental data, as shown in Figure 3, a 150 mm section of the sample was cut in the middle of the pipe fitting. The sample was soaked in water at the same temperature as that used in the experiment for 70 min, the sample was clamped with a fixture, and the machine was started to begin testing.

## 3. Results and Discussion

### 3.1. Effect of the Processing Method on the Wall Thickness and Uniformity of Pipes

**Figure 4:**
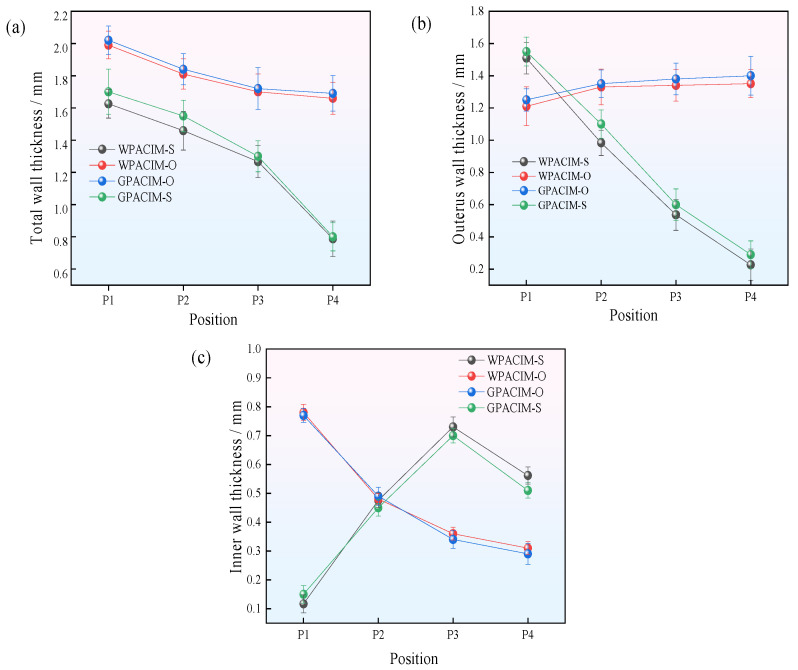
The residual wall thickness of the four processing pipes: (**a**) Total wall thickness; (**b**) Outer wall thickness; (**c**) Inner wall thickness.

The above measurement method was used to measure the wall thickness of pipes formed by different injection molding processes. As shown in Figure 4a, the total wall thickness of the four injection molding process fittings gradually decreases along the axial direction. This is because the amount of melt gradually decreases compared with the initial penetration of the projectile or fluid medium during the molding process, resulting in a decrease in penetration resistance. This phenomenon allows the projectile to drag more molten material forward. Therefore, as the penetration cross-section increases, the total wall thickness of the pipe gradually decreases. The total wall thickness of the short-shot method pipe fittings is thinner than that of the overflow method pipe fittings. However, the overall wall thickness variation trend of the short-shot method pipe fittings differs significantly from that of the overflow methods. This phenomenon may be because the outer layer of molten material only fills part of the cavity during the short-shot molding process. As the inner layer of molten material and the projectile push, the outer layer of molten material is squeezed forward, forming a new outer wall. There will be a significant difference in the total wall thickness size between the initial filling section and the newly formed section of the pipe melt, so the total wall thickness of the pipe produced by the short-shot method will decrease more significantly. During the penetration process, the newly formed section of the pipe has a high melt temperature and good fluidity. However, the pressure of the fluid medium remains constant, resulting in a further increase in the penetration cross-section. Therefore, compared with the short-shot method, the overflow method results in a greater total pipe wall thickness.

Figure 4b shows that the outer wall thickness of the W-PACIM-O and G-PACIM-O process fittings gradually increases along the axial direction of the fittings. At the same time, it remains almost unchanged at positions P2 to P4. This phenomenon is due mainly to the influence of two types of interactions during the pipe penetration process by the overflow method. On the one hand, the temperature of the melt is initially high, the amount is large, and the viscosity is low, which improves the melt flow. Fluid media and projectiles can push more molten material forward, resulting in a thinner outer wall for the pipe. On the other hand, during the continued penetration process, owing to the intensified heat exchange between the mold wall, fluid medium, and the projectile and melt, the temperature of the melt gradually decreases, and the viscosity increases, increasing the outer wall thickness of the pipe. At positions P1 to P2, the latter has a more significant impact than the former, increasing the outer wall thickness of the pipe. However, at positions P2 to P4, the change in outer wall thickness is not significant because of the comparable effects of the two influences.

In contrast, the outer wall thickness of the pipe formed by the short-shot method gradually decreases along the axial direction, with a reduction of 85%. This phenomenon is attributed mainly to the fact that in the short-shot method, the outer melt continues to move forward in the form of a fountain flow, filling the entire cavity. During this process, after a specific delay in gas/water injection, the newly formed outer layer of molten material comes into contact with the mold wall and undergoes heat exchange. Therefore, compared with the initial filling stage, the contact time between the outer layer melt and the mold wall is shorter, resulting in a lower viscosity of the newly formed outer layer melt. This phenomenon reduces the penetration resistance of the inner layer melt in the outer layer melt, allowing the inner layer melt to carry more of the outer layer melt forward, ultimately gradually thinning the outer layer wall thickness.

Figure 4c shows that the inner wall thickness of the overflow method pipe gradually decreases along the flow direction. This phenomenon indicates that during the penetration process, the penetration length of the fluid and projectile continues to increase, and the penetration resistance gradually decreases. However, the pressure of the fluid medium remains constant, resulting in a further increase in the penetration cross-section; therefore, the inner wall thickness of the pipe decreases. In addition, the inner layer melt is far from the mold wall, resulting in poor heat exchange efficiency, minimal temperature changes, low viscosity of the inner layer melt, and good fluidity. When the fluid penetrates, it can push more of the inner layer melt forward. The introduction of projectiles can isolate the direct contact between the melt and the fluid medium so that the fluid medium only plays a role in providing penetration power during the forming process, thus reducing the influence of the fluid medium itself on the inner layer melt. Therefore, the reduction in the inner wall thickness of pipes formed from different media overlaps.

Unlike the trend of the inner wall thickness variation in the overflow method fittings, the inner wall thickness of the short-shot method fittings shows an increasing trend in the P1–P3 section and a decreasing trend in the P3–P4 section (end). The reason is that, compared with those in the overflow method, the filling amounts of the inner and outer layer melts in the short-shot method are relatively small. In contrast, the resistance of the inner layer to the outer layer melt during penetration is relatively reduced. The change in penetration resistance has a more significant effect on the inner layer melt penetration cross-section than on the solid projectile penetration cross-section. Therefore, thinning of the outer layer wall is evident, resulting in an increasing trend in the inner layer wall thickness along the flow direction. In the P3–P4 section, to ensure the penetration length in the short-shot method, the amount of molten injection was controlled, resulting in a smaller amount of molten material at the end. Therefore, the residual wall thickness of both the inner and outer layers tended to decrease.

Regardless of the molding method used, the wall thickness of pipes formed with gas is greater than that of pipes formed with water as the fluid medium. The reason is that there are significant differences in mass and heat transfer when different fluid media come into contact with the melt during the molding process. The thermal conductivity of water is 40 times greater than that of gas. During the penetration process, there is intense heat exchange between the water and the melt, resulting in high cooling efficiency, reduced melt viscosity, and decreased fluidity. Moreover, water is incompressible and can continuously exert pressure on the melt on both sides [18]. Ultimately, this leads to a gradual decrease in the total wall thickness of the fittings in the WPACIM process. Compared with water, gas is compressible. When it penetrates, the molten material on both sides compresses the gas, resulting in a smaller hollow cross-section and an increase in the wall thickness of the pipe.

When using the same molding method but different fluid media, the difference in the residual wall thickness of the pipe fittings is relatively small. This phenomenon occurs because during the pipe-forming process, the fluid medium provides penetration power for the projectile, and the size of the projectile is fixed and in direct contact with the melt. Therefore, the residual wall thickness of the pipe fittings has been effectively controlled.

### 3.2. Effect of the Processing Method on Pipe Roughness

**Figure 5:**
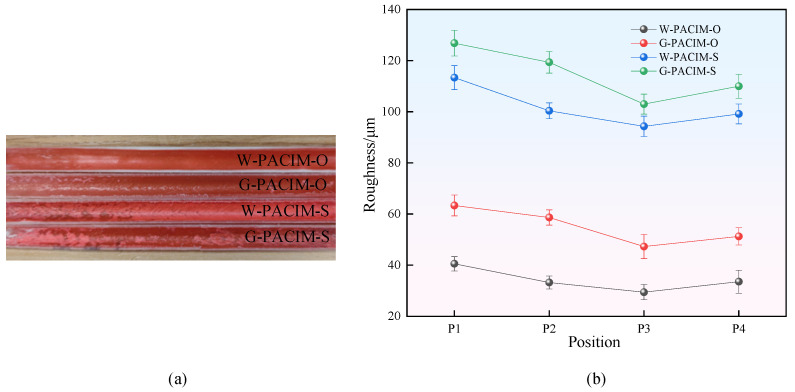
Three-dimensional physical drawings and bar charts of different processes: (**a**) Different process pipe fittings; (**b**) Bar chart.

According to Figure 5, the inner wall surfaces of the pipes prepared by four different processes all exhibit a roughness variation trend of first decreasing and then increasing along the direction of melt flow. The cause of this phenomenon may be due to the presence of turbulent vortices in the fluid medium during the initial stage, resulting in a relatively rough inner wall surface of the melt. As the fluid medium continues to move forward, its flow state gradually becomes stable, and the penetration process also becomes smoother, thereby making the inner wall of the pipe gradually smoother. However, when the fluid medium moves to the tail of the mold cavity, due to the significant difference in size between the overflow cavity and the mold cavity, the melt will accumulate, and the fluid medium will also experience some degree of fluctuation. These factors work together to increase the roughness of the inner wall surface of the pipe fittings again.

Figure 5 shows that, compared with the short-shot method pipe fittings, the inner wall surface of the overflow method pipe fittings appears smoother. This phenomenon may be due to the presence of “primary penetration” of the filling stage fluid in the melt and “secondary penetration” caused by the cooling shrinkage of the melt during the holding pressure cooling stage in F-PACIM-S, which affects the surface quality of the product. In the overflow method, owing to the presence of an overflow cavity, fluid penetration can be carried out once the melt fills the mold cavity. Excess melt is squeezed into the overflow cavity, eliminating the phenomenon of secondary penetration and improving the quality of the inner wall of the pipe fitting.

When the same injection method is used, the inner wall surface of the pipe with water as the fluid medium is smooth. This phenomenon occurs because, compared with gas, water has greater incomprehensibility and a higher specific heat capacity [19]. Therefore, a higher cooling efficiency results in lower crystallization and a smaller spherulite size of the semicrystalline melt, thereby reducing the surface roughness of the pipe’s inner wall [20]. High-pressure gases have comprehensibility. When high-temperature melts are pushed, some gases merge into the melt. As the pressure and melting temperature decrease, supersaturated gases precipitate from the melt, resulting in white bubble spots on the inner surface of the pipe. This phenomenon leads to an increase in roughness on the inner surface of the pipe fittings.

### 3.3. Effects of the Processing Method on the Glass Fiber Orientation in the Pipes

**Figure 6:**
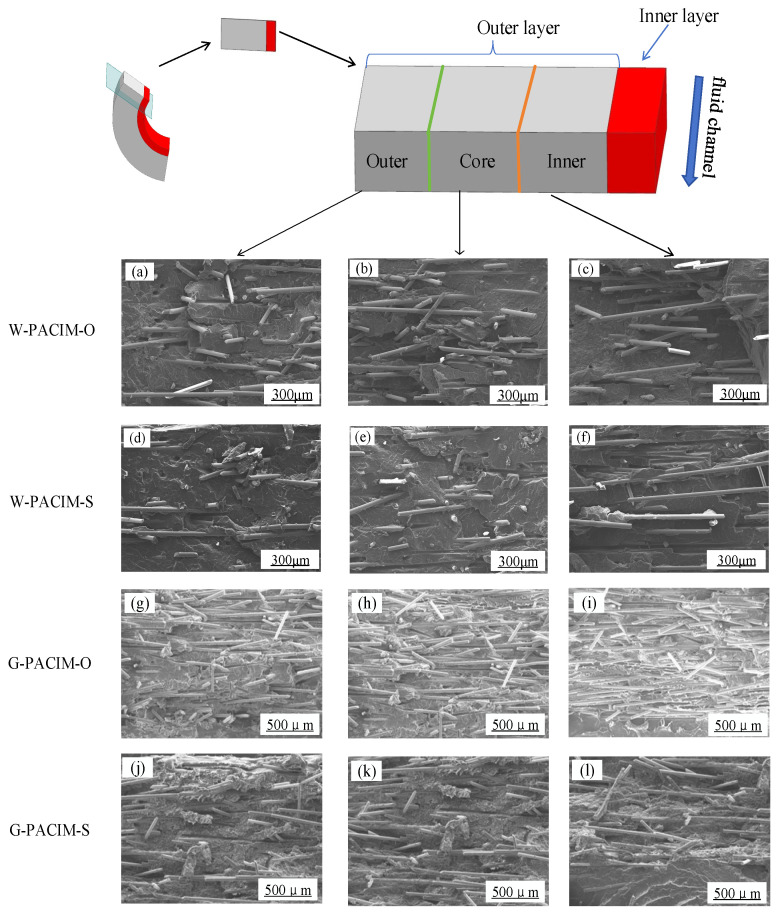
Observation map of the fiberglass orientation for different processes. (SEM magnified images of the outer near wall layer, middle layer, and outer near boundary layer of each process from left to right).

Different processing methods can affect the axial distribution and orientation of glass fibers in the external melt, resulting in changes in the mechanical properties of the pipe fittings [21]. In order to ensure the accuracy of the experimental results, the fiber orientation at the same position was measured multiple times by scanning electron microscopy, and the chemical compatibility or adhesion between the glass fiber and the polymer matrix was ensured to be the same under different processing conditions. The results, as shown in Figure 6a–l, are enlarged views of the local area of the outer wall thickness.

By comparison, the pipes produced via the short-shot method and overflow method are generally divided into three regions in the outer section: the mold layer, the core layer, and the near-boundary layer. The distribution patterns of glass fibers in different regions of the two injection methods are similar. Overall, the orientation of the glass fibers at the core layer is the best, followed by that at the near boundary layer, whereas the orientation degree of the mold layer is the lowest, and the shape distribution is relatively disordered. However, there are also significant differences between the two injection methods. The glass fiber density of the cross-section of the pipe made via the overflow method is significantly greater than that of the short-shot method. The bonding effect between the PP matrix and glass fibers is greater, whereas the peeling phenomenon between the glass fibers and PP resin is more severe with the short-shot method. Second, there are fewer holes and more small grooves on the cross-section of the pipe formed by the overflow method, indicating that the distribution of glass fibers in the overflow method is mainly parallel to the direction of melt flow, whereas, in the short-shot method, the distribution of glass fibers is mainly perpendicular to the direction of melt flow.

In addition, Figure 6 shows that the degree of glass fiber orientation in the overflow method is generally greater than that in the short-shot method. The main reason is that in the pipe fittings formed by the short-shot method, the outer wall thickness is relatively thin (as shown in Figure 4 and previous analysis), and the heat exchange effect of the mold wall is more muscular, resulting in a faster cooling rate and increased viscosity of the outer melt, thereby reducing the activity of the glass fiber. Compared with the short-shot method, the overflow method results in most glass fibers being perpendicular to the flow direction of the melt. Therefore, when a sample undergoes brittle fracture in the circumferential direction, the glass fibers are more easily extracted from the PP matrix, resulting in the formation of more holes. This also reflects the poor orientation of the glass fibers in the flow direction of the short-shot method pipe fittings.

For both the short-shot method and the overflow method, the glass fiber orientation degree of the core layer is the worst. This phenomenon occurs because when the mold cavity is filled with melt, the outer layer near the mold wall first contacts the mold wall and generates a shear force (opposite to the direction of the melt flow) with the mold wall. This force gradually decreases from the outer layer near the mold wall to the outer layer near the interface, resulting in the highest degree of glass fiber orientation at the outer layer near the mold wall. However, when the inner layer melts and the projectile penetrates through the outer layer melt, the outer layer melt experiences shear forces opposite to those in the previous stage (along the flow direction of the melt). The shear force gradually decreases from the outer layer near the interface to the outer layer near the mold wall. Owing to the two opposite shear forces, the degree of glass fiber orientation is lower in the middle position.

In addition, there are differences in the influence of various fluid media on the axial orientation of glass fibers. Owing to the rapid decrease in melt temperature and solidification caused by water, the orientation of the glass fibers in the axial direction is more effectively maintained. Therefore, the orientation of the fiberglass along the axial direction in pipes with water as the fluid medium is better than that in pipes with gas as the fluid medium.

### 3.4. Effects of the Processing Method on the Maximum Instantaneous Burst Pressures of Pipes

**Figure 7:**
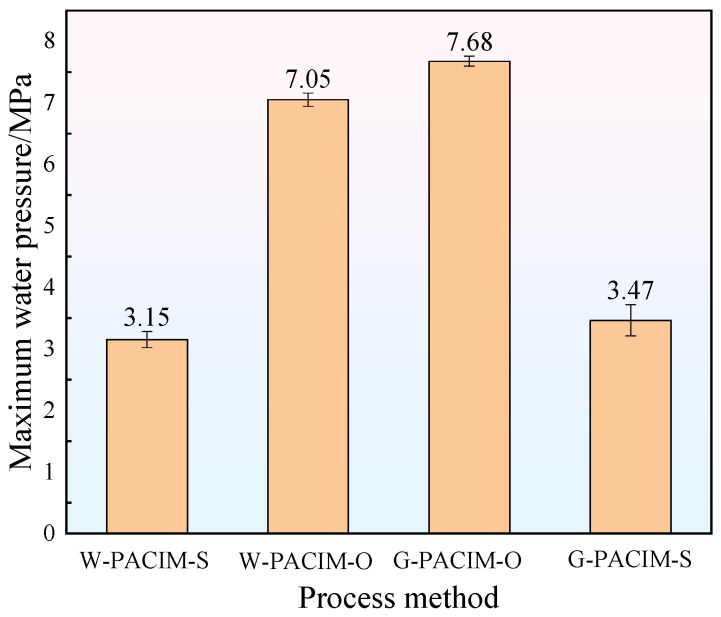
Maximum instantaneous burst pressure of pipe fittings.

For the components of polymer composite materials, the mechanical properties of the components are mainly used as an important standard to measure the quality of the components in various industrial fields. But for pipe fittings, the main focus is on exploring their pressure resistance performance. To this end, the maximum instantaneous burst pressure of pipes formed by four different process methods was tested using a pipe pressure tester at a water temperature of 20 °C. The test results are shown in Figure 7.

According to Figure 6, the G-PACIM-O pipe fittings have the best pressure resistance performance, whereas the W-PACIM-S pipe fittings have the worst. When a pipe is blasted, it experiences an axial fracture, which indicates that when the projectile and fluid medium penetrate, the orientation of the glass fibers in the melt changes, the circumferential strength increases, and the axial strength decreases. Therefore, under an external force, cracks can form in the axial direction. This phenomenon is influenced mainly by the pipe wall thickness, uniformity, and glass fiber orientation.

As shown in Figure 4, the total wall thickness, outer wall thickness, and inner wall thickness of the overflow method pipe fittings are greater than those of the short-shot method pipe fittings. Assuming the same material parameters and without considering other influencing factors, a thicker wall indicates a greater ability to resist water pressure surges and better pressure resistance. By calculating the uniformity of the wall thickness of the pipe fittings, it can be seen that the uniformity of the short-shot method pipe fittings is not significantly different from that of the overflow method except in the P4 section. Its uniformity in the P1–P3 section is worse than that of the overflow method. This phenomenon indicates that the short-shot method pipe fittings have a smaller wall thickness and a more significant deviation in wall thickness at the same position than the overflow method does. At the same position, the wall surface of the short-shot method pipe fittings is weaker than that of the overflow method, and the pressure resistance performance further decreases. Figure 5 shows that the orientation of the glass fibers in the short-shot method pipe fittings along the direction of the melt flow is poor, and most of the glass fibers are perpendicular to the flow direction, making them prone to mechanical defects. In the overflow method, the orientation of the glass fibers is better, and many glass fibers are oriented at a certain angle to the flow direction, forming a tight, three-dimensional cross structure. When subjected to external force impact, the dispersion effect of the external force is better, and the pressure resistance performance is good.

In addition, the pressure resistance performance of pipes made in a gas medium is slightly better than that of pipes made in a water medium, but the numerical difference is negligible. From a macroscopic perspective (see Figure 4), the difference in wall thickness and thickness uniformity between the two process pipes is minimal, and some parts even overlap. At the microscopic level (as shown in Figure 6), introducing pellets during the injection molding process results in little difference in the orientation of the outer glass fibers between the two processes, thus resulting in similar pressure resistance performance.

## 4. Conclusions

This article focuses on analyzing the wall thickness, wall thickness standard deviation, inner surface roughness, glass fiber orientation, and pressure resistance of the pipes. On the basis of the comparison and analysis of the experimental results, the following conclusions can be drawn:

The total wall thickness of pipes formed by the overflow and short shot method decreases along the flow direction, but the overflow method decrease is insignificant. In addition, the circumferential uniformity of the overflow method pipe fittings is significantly better than that of the short-shot method. When formed via the same method, the fluid medium has a relatively small effect on the wall thickness of the pipe fittings. The degree of fiber orientation along the flow direction in the outer layers of each pipe demonstrates a discernible trend of increasing from the near-mold wall layer to the outer core layer and then to the near-interface layer. Compared with overflow method fittings, short-shot method fittings have poorer overall glass fiber orientation and are mainly perpendicular to the flow direction. The pressure resistance performance is related mainly to the residual wall thickness, the uniformity of the pipe fittings, and the distribution of the micro-glass fiber morphology orientation. The performance of pipe fittings formed by the short-shot method is worse than that of the overflow method. W-PACIM-O process pipes have more significant advantages when the material applicability and pressure resistance requirements are not high.

## Figures and Tables

**Table 1:** Forming parameters of the four processes.

Process/Material Parameters	Value			
	G-PACIM-S	G-PACIM-O	W-PACIM-O	W-PACIM-S
Outer melt temperature (°C)	250			
Inner layer melt temperature (°C)	220			
Inner layer melt injection delay time (s)	3			
Outer layer melt injection pressure (MPa)	5.5			
Inner layer melt injection pressure (MPa)	9			
Injection delay time (s)	5			
Injection pressure (MPa)	7			
Die temperature (°C)	50			

## Data Availability

Data are contained within the article.
